# Supplementary material for: Demonstrating an approach for evaluating synthetic geospatial and temporal epidemiologic data utility: Results from analyzing >1.8 million SARS-CoV-2 tests in the United States National COVID Cohort Collaborative (N3C)
Source: medRxiv. 2021 Jul 8:2021.07.06.21259051. Preprint. [Version 1] doi: 10.1101/2021.07.06.21259051 (PMC8282114; doi:10.1101/2021.07.06.21259051)

SUPPLEMENT

Figure Titles:

S2: Distribution of total tests per zip code in original data which were censored within the synthetic data

S3: MDClone data synthesis workflow

Table S1: Zip code month pairs' synthetic error central tendencies and counts stratified by indicator and bin size.

indicator	Number of zip codes stratified by month	Bin value original count	Synthetic Error mean (stdev)	Synthetic Error median (IQR)
Tests	33328	0-19	-0.14 (±1.9)	0 (2)
Tests	5283	20-49	-0.54 (±3.31)	-1 (5)
Tests	2697	50-99	-0.4 (±4.11)	0 (5)
Tests	2230	100-249	-0.28 (±5.17)	0 (6)
Tests	1102	250-1705	-0.59 (±7.29)	0 (9)
Positives	26707	0	0.07 (±0.37)	0 (0)
Positives	6499	1	-0.55 (±0.92)	-1 (1)
Positives	6264	2-5	-0.78 (±1.76)	-1 (2)
Positives	4715	6-49	-0.59 (±2.63)	-1 (3)
Positives	455	50-520	-1.13 (±4.22)	-1 (5)
Admissions	37963	0	0.04 (±0.25)	0 (0)
Admissions	3837	1	-0.43 (±0.82)	-1 (1)
Admissions	2078	2-4	-0.66 (±1.42)	-1 (2)
Admissions	499	5-9	-1.37 (±2.29)	-1 (3)
Admissions	263	10-80	-2.16 (±3.33)	-2 (4)

Figure S2

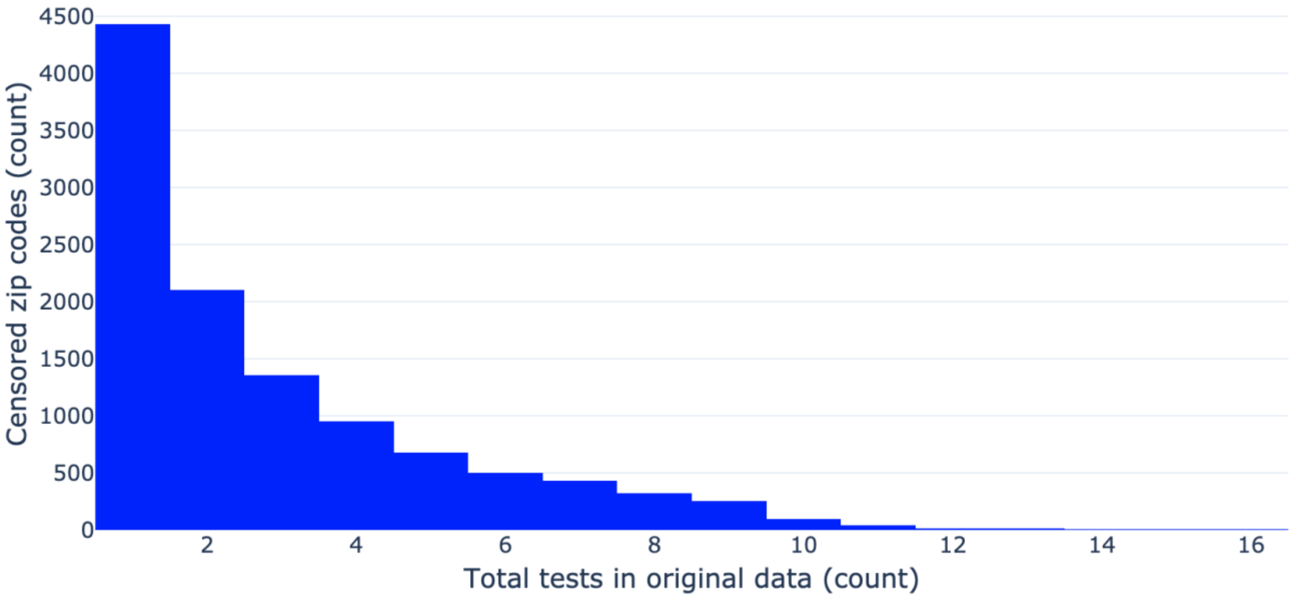

Figure S3

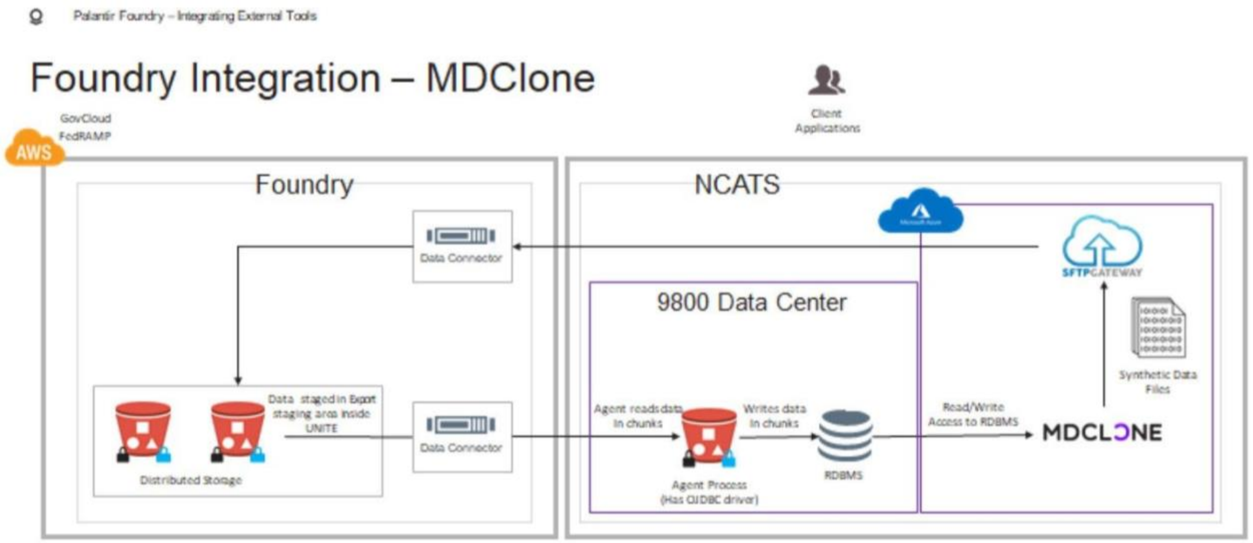

Supplement: Supplement 1 [file media-1.pdf]
